# OptimDase: An Algorithm for Predicting DNA Binding Sites with Combined Feature Encoding

**DOI:** 10.1007/s12539-025-00704-8

**Published:** 2025-06-10

**Authors:** Zhendong Liu, Jun S. Liu, Dongqing Wei, Rongjun Man, Jiamin Jiang, Bofeng Zhang, Liping Li, Zhiyong Zhao

**Affiliations:** 1 School of Computer and Information Engineering, Shanghai Polytechnic University, Shanghai, 201209 China; 2 Department of Statistics, Harvard University, Cambridge, 02138 USA; 3 State Key Laboratory of Microbial Metabolism, Joint International Research Laboratory of Metabolic & Developmental Sciences, and School of Life Sciences and Biotechnology, Shanghai Jiao Tong University, Shanghai, 200030 P.R. China; 4 Department of Otolaryngology, Shandong Provincial Hospital Affiliated to Shandong First Medical University, Jinan, 250021 China; 5 Department of Medical Imaging, Shandong Provincial Hospital Affiliated to Shandong First Medical University, Jinan, 250021 China

**Keywords:** DNA binding site, Machine learning, optimum decision-making, Site-specific recombination, combined feature encoding

## Abstract

Identifying DNA binding sites remains a critical task in bioinformatics, with applications ranging from gene regulation studies to drug design. Although progress has been made in computational techniques, we still face challenges such as data complexity and prediction accuracy. In this paper, we introduce OptimDase, a new algorithm. It integrates feature encoding with optimum decision-making frameworks to improve DNA binding site prediction. OptimDase integrates multi-scale scanning and feature selection strategies, making it highly effective for both classification and regression tasks. Our experiments demonstrate that OptimDase achieves superior performance with an accuracy of 0.8943 in classification tasks and an RMSE of 0.0054 in regression tasks, outperforming existing algorithms in key evaluation metrics. These results highlight OptimDase’s portability and robustness, making it an effective solution for identifying DNA binding sites and advancing the applications of drug design.

**Graphical Abstract:**

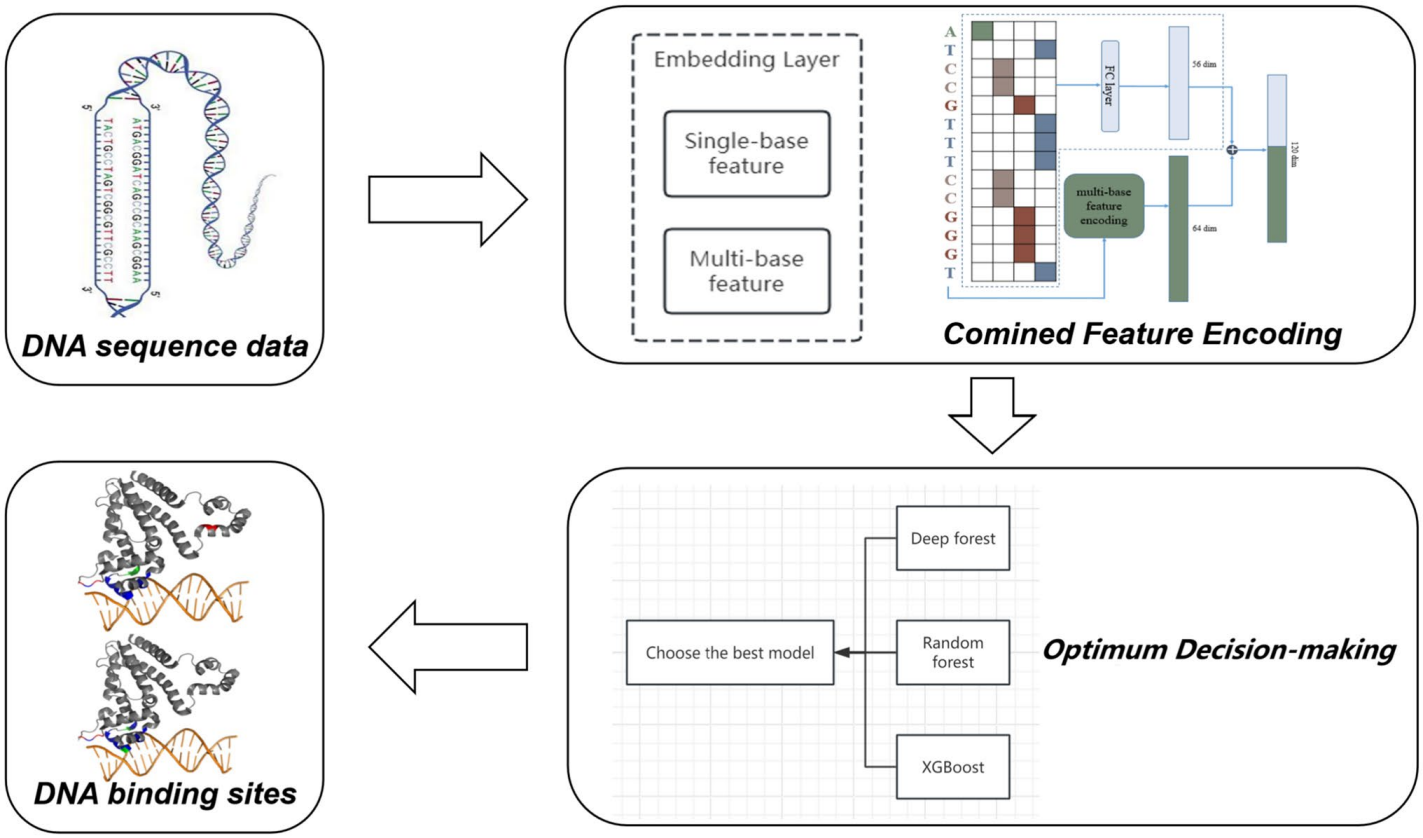

## Introduction

DNA–protein interactions are fundamental to various cellular processes, including growth, differentiation, and gene regulation [1]. These interactions are critical for both the preservation and expression of genetic information [2, 3], making the identification of DNA binding sites essential for grasping these biological mechanisms [4]. While traditional experimental methods like Chromatin Immunoprecipitation Sequencing (ChIP-seq) have been instrumental in detecting these binding sites [5, 6], they often require substantial resources and are limited by the availability of specific reagents, such as antibodies for transcription factors.

In light of these challenges, computational methods have emerged as a valuable complementary approach to identify DNA binding sites, particularly for transcription factors that typically interact with short sequences of 5 to 20 base pairs [7]. Early computational techniques primarily utilized motif-based approaches [8] and position weight matrices (PWMs). These strategies often struggled to capture the complexity of DNA–protein interactions [9, 10]. Factors beyond simple binding motifs play a significant role in this complexity. For instance, neighboring sequences, GC content [11], and the three-dimensional structure of DNA all contribute to binding specificity [12]. To enhance prediction accuracy, recent models have integrated additional features derived from ChIP-seq data [13, 14].

On the other hand, recent studies on site-specific recombination have highlighted the significant role of attCs, known for their distinct structural properties and sequence constraints that are crucial for establishing an efficient system for site-specific recombination [15, 16]. Traditionally, the identification of attCs has relied heavily on biochemical assays and conventional bioinformatics methods. However, advancements in these fields often face limitations due to technological constraints, time pressures, and budgetary issues. Biochemical techniques such as fluorescent labeling and electrophoresis have been commonly used to predict DNA recombination sites [17, 18].

Nowadays, machine learning is widely used by researchers to address and solve predictive problems across various fields and interdisciplinary areas [19–21]. In terms of performance, it offers the benefits of a brief training duration and a reduced risk of overfitting; most importantly, it outperforms traditional computational methods in terms of prediction results. On the other hand, due to the massive production of biological data, traditional biological data analysis methods struggle to cope with the increasingly complex and voluminous biological sequence data. In response to this dilemma, deep learning has gradually been applied in biological analysis. In recent advancements, scholars have proposed FCNA and AgentBind algorithms to detect transcription factor binding site (TFBS) motifs [22, 23]. However, it has also been observed that CNNs have difficulty in capturing long-range dependencies across different positions in DNA sequences. Furthermore, in deep learning models like DeepGRN and DeepSTF, incorporating attention mechanisms to enhance performance has become a widely adopted strategy [24, 25]. Alipanahi et al. [26]. proposed DeepBind founded on CNNs, achieving automatic feature extraction and prediction. DeepBind was more effective than other complex methods at the time of its proposal, but it performed less well than expected when dealing with complex genomic regions. DanQ achieved simultaneous prediction of 690 TFBSs [27]. It has portability and can also predict protein modifications and open chromatin region (DHS) sites. Clearly, mainstream machine learning algorithms still have some shortcomings, such as less than ideal accuracy, long computation times, and overfitting issues. Despite these challenges, machine learning algorithms remain the top-performing techniques for identifying TFBSs and hold significant capability [28].

In this study, we propose an algorithm, OptimDase, for identifying DNA interaction sites because identifying DNA binding sites is a meaningful and difficult task. OptimDase is based on the optimum decision-making, which is implemented by XGBoost, Deep Forest, and Random Forest algorithms. These three algorithms excel in tasks due to their unique strengths that enhance model performance. Deep Forest is suitable for hierarchical feature learning in small datasets, Random Forest reduces overfitting and performs well in feature selection, while XGBoost improves model accuracy through gradient boosting, making it ideal for large-scale data. The dataset of OptimDase was from human chromosome 1 transcription factor SP1 and attCs, and contrasted the effectiveness with conventional deep learning algorithms. According to the study, the predictive features of OptimDase are significantly better than those of other existing algorithms. Importantly, it tackles the shortcomings of current algorithms that are primarily concerned with low prediction accuracy. Moreover, we analyzed the critical aspects of DNA binding sites, providing a foundation for later studies in the identification of DNA binding sites. Furthermore, this study built the model using the Python programming language on the Linux operating system, ensuring that OptimDase has strong robustness and portability.

## Results

OptimDase is based on machine learning algorithms and integrates optimization strategies. This algorithm can be utilized to pinpoint DNA binding sites. OptimDase first judges the task type and then selects three different models according to the task type. After using the Optuna framework for parameter optimization, the training data is used. Further, the trained model receives the test data as input to evaluate the performance, which is selected as the final model when a model is determined to be the optimal model. The integrated algorithm of OptimDase includes Random Forest, XGBoost, and Deep Forest algorithms which is inspired by varied granularity scanning. In this section, we undertake the comparison of our algorithm with several prevalent methods currently employed for DNA site prediction. Our experimental analysis leverages data regarding TFBSs. In Sect. 2.1, we explore the benefits of combined feature encoding in contrast to single base encoding. Section 2.2 presents the comparative results of OptimDase against traditional machine learning approaches. Furthermore, Sect. 2.3 identifies main attributes and their associated weights within the DNA site databases. It is essential to emphasize that the results derived from the data utilized a five-fold cross-validation methodology.

### Comparison with Single Base Encoding

The approach to identifying TFBSs in DNA sequences goes beyond simply analyzing individual nucleotide features. It is crucial to consider how neighboring nucleotides interact, as these interactions play a significant role in the binding process. To explore this concept, we conducted a study comparing two types of models: those that rely only on single nucleotide characteristics and those that take into account the interactions among multiple nucleotides. Our experimental results clearly demonstrated that the models which integrated interactions among several nucleotides achieved much higher accuracy, F1 scores, and area under the curve (AUC) metrics than those based solely on single nucleotide data. This trend was consistent across all algorithms we evaluated. We focused on five key algorithms that delivered the best results. As shown in Table 1, every algorithm experienced an uptick in predictive accuracy, typically between 1% and 3%. These findings suggest that a more holistic approach to feature representation, which captures the complexities of DNA sequences, is far more effective at uncovering critical biological information.

**Table 1:** Comparison of the different encoding methods for characteristics in various algorithms

Metric	Encoding method	Random forest	KNN	Deep forest	Adaboost	LightGBM
Accuracy	Single base encoding	0.8813	0.8513	0.8659	0.8532	0.8538
	Combined feature encoding	0.8855	0.8514	0.8834	0.8579	0.8794
F1-score	Single base encoding	0.8718	0.8461	0.8550	0.8441	0.8545
	Combined feature encoding	0.8813	0.8551	0.8864	0.8610	0.8785
AUC	Single base encoding	0.9035	0.8758	0.8940	0.8856	0.8920
	Combined feature encoding	0.9136	0.8819	0.9183	0.9001	0.9088

### Comparison with Machine Learning Models

**Fig. 1:**
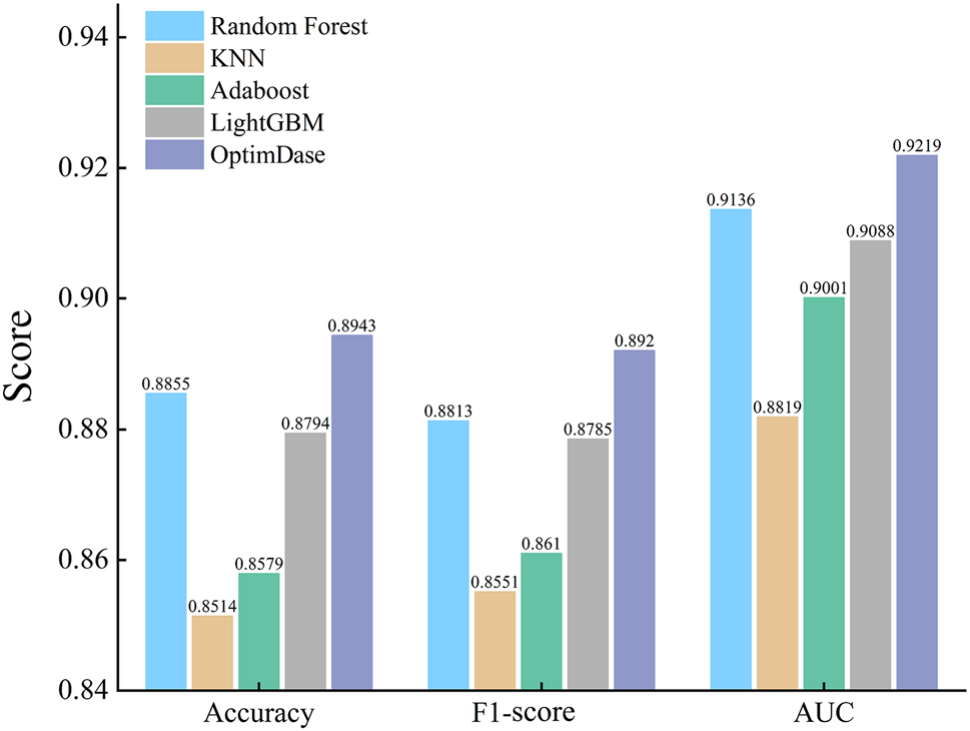
Comparison of the prediction performance results for all algorithms on the TFBS dataset

OptimDase integrates Random Forest, XGBoost, and Deep Forest. Different types of datasets have distinct characteristics and complexities. Therefore, using different algorithms for comparison can better adapt to the specific features of each dataset, resulting in a more accurate performance evaluation. Certain algorithms may perform better on specific datasets, while others may excel in different contexts. To highlight the varying scenarios of dataset handling, we selected different comparison algorithms to demonstrate the robustness and broad applicability of OptimDase under different conditions. This subsection includes a comparison with other existing machine learning algorithms like AdaBboost and LightGBM. The reason for choosing these machine learning methods for comparison is that they have a broad application foundation and reference value in our study. The hyperparameters of all algorithms are obtained using the Optuna framework. Figure 1 shows the prediction performances of the common algorithms applied to the TFBS dataset.

**Fig. 2:**
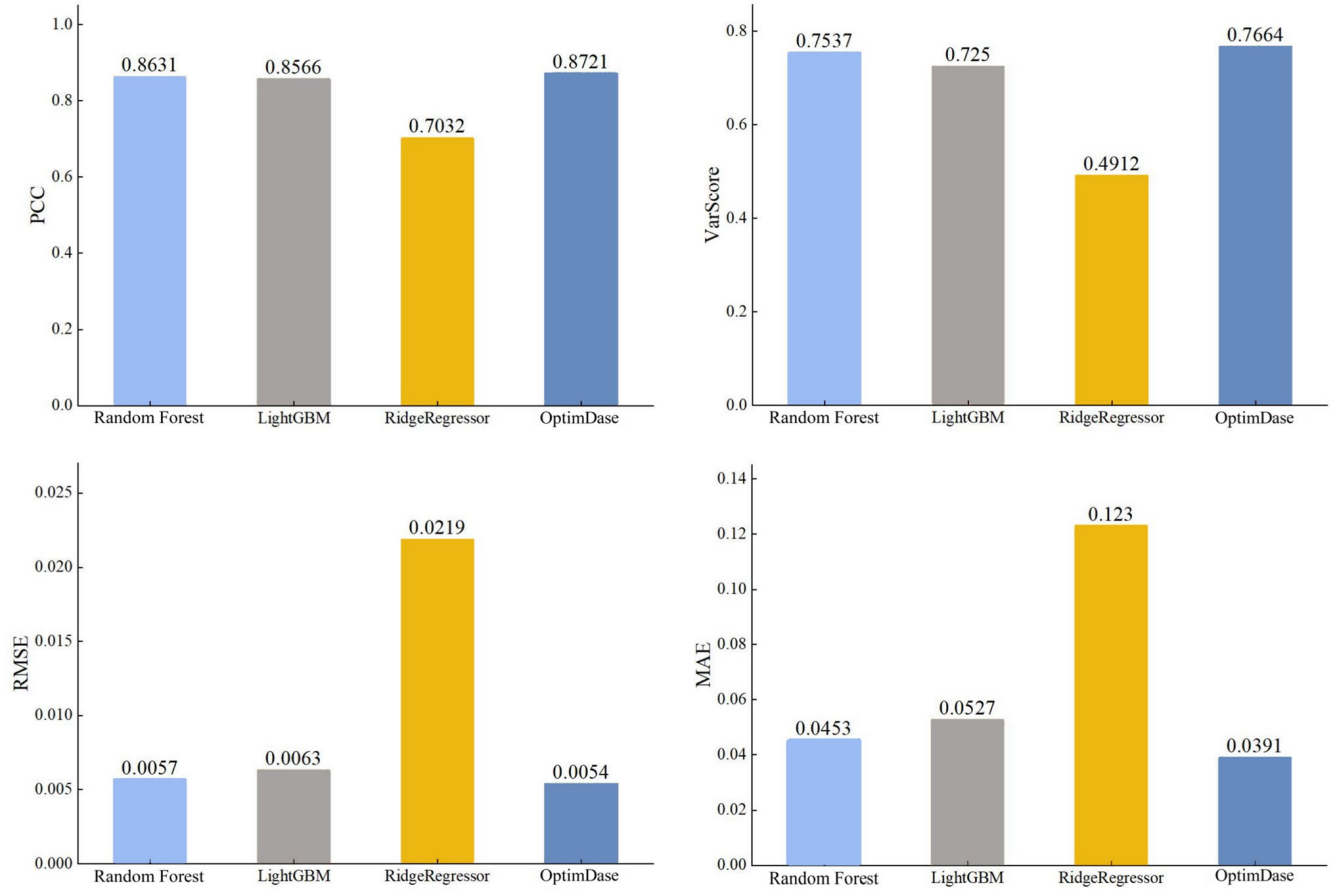
Comparison of the prediction performance results for all algorithms on the attC dataset

Figure 2 displays the prediction performances of the common algorithms applied to the attC dataset, showing that OptimDase uniformly outperformed all other algorithms for predicting DNA binding sites. While the higest PCC and VarScore values among all the algorithms suggest that OptimDase can better reflect changes in the data set than others. The lowest RMSE and MAE scores demonstrate that OptimDase is also good at predicting at the actual values. The uniform improvements of OptimDase over other methods for TFBS prediction warrant further AI-based investigations of protein-DNA binding mechanisms.

### Aggregating Significant Feature Weights

The robust approach of feature significance assessment facilitates algorithms in prioritizing the most impactful characteristics, thereby boosting their overall effectiveness. To establish weight scores for each feature, we constructed multiple decision trees and evaluated the Gini index across various nodes within these trees. The weight scores assigned to each feature are normalized to fall within the range of 0 to 1, ensuring that they sum to 1. Features with higher weight scores are viewed as more crucial to the model’s predictive capabilities.

Our algorithm captures the calculated weights of each locus data in the independent dataset through the varied granularity sliding strategy that employs weighting, which is applied to the different sample datasets. The weights are then sorted in descending order to create Table 2. Therefore, we list the features and weights of the attC locus and SP1 dataset separately to better demonstrate the workings of the algorithm. The first two columns highlight the most significant features based on weights in the attC dataset. This selection encompasses both global and fundamental traits, indicating that the recombination ratio at attCs has been influenced by the complex interplay of several significant traits. The remaining columns present the leading characteristics from the SP1 dataset along with their respective weights. In this case, the most important features arise from a multi-base representation, emphasizing the critical role that inter-base signal characteristics play in enhancing the accuracy of classification predictions. This illustrates the necessity of incorporating a diverse array of features to fully understand and improve the model's predictive power.

**Table 2:** The top 20 scores of the feature weights

attCs		TFBSs	
Feature name	Weight	Feature name	Weight
Boltz_dG_u	0.045005	GCG	0.094719
pos_entr_37_u	0.042199	CGC	0.082571
pos_entr_18_u	0.042031	GCC	0.061100
pos_entr_44_u	0.039102	GGC	0.058620
base_54	0.027229	CCG	0.058140
base_45	0.016903	CGG	0.057638
bp_proba_18_46_u	0.013347	CCC	0.031515
bp_proba_17_47_u	0.013075	GGG	0.024249
bp_proba_9_55_u	0.011480	TAA	0.023230
pfold	0.011212	TTA	0.022269
pos_entr_45_u	0.011182	ATT	0.021181
pos_entr_30_u	0.010561	AAT	0.020856
bp_proba_15_48_u	0.008583	TAT	0.015082
pos_entr_38_u	0.008519	ATA	0.014472
pos_entr_39_u	0.007891	AAA	0.012274
MFE_freq_u	0.007707	TTT	0.012183
pos_entr_63_u	0.007095	GTA	0.009278
bp_proba_17_48_u	0.007057	ATG	0.009041
dG_ratio_BOT_TOP_u	0.005546	CAT	0.008764
bp_proba_28_34_u	0.005458	TAC	0.008354

## Discussion

In our research, we explored the use of our own encoding method to identify important characteristics from the context of DNA bases while analyzing sequence data related to DNA binding sites. Our experiments showed that the best results came from representing features in triplets, which aligns with the biological principle that amino acids are formed by sequences of three bases. Moreover, when we examined the significance of different features, we found that the most impactful ones were derived from our multi-base encoding method. This finding reinforces the idea that employing a multivariate feature encoding strategy is crucial for enhancing our analysis of DNA sequences. Overall, our work highlights the importance of combining various encoding techniques to gain a more nuanced understanding of DNA binding interactions.

We conduct a contrastive summary of the experimental outcomes with currently popular algorithms. The final outcomes reveal that OptimDase validly enhances the prediction precision on most datasets. In both regression and classification tasks, OptimDase has outperformed other considered methods. We expect that the algorithm is robust enough and can be easily adapted to other datasets, such as the prediction of the frequency of other DNA recombination sites or identification of other DNA binding sites. In addition, we also analyzed the feature weights of the SP1 dataset and the attC dataset, which improve the precision of identifying SP1 and the recombination frequency of attCs, respectively.

Although OptimDase performs well for small sample data, a limitation of the method is that at the moment the algorithm is not suitable for large datasets. When a very large training set is available, a DNN-based method may be more preferrable. In addition, the shape and structure of DNA, along with its sequence, impact DNA binding activities [29].

## Materials and Methods

### Data Preparation

#### Data Collection

Our study evaluated OptimDase and compared it with several leading competitors using two well-known public datasets focused on the attCr0 mutation dataset along with TFBS dataset. These collections are widely used for comparative analysis of models due to their accessibility. The attCr0 mutant library consists of sequences, each containing the stable attCr0 region. This design is crucial for assessing the accuracy of model predictions at DNA recombination sites. In our primary analysis, we selected sequences from the attCr0 region that contained a dataset of 14,569 attC mutants.

Furthermore, to further validate our model's ability to predict DNA binding sites, we obtained TFBS dataset from Kaggle.com, with a particular focus on TFBSs located on human's first chromosome. This collection comprises 2,400 entries, each providing the DNA strand with 14 bases along with the relevant tag. TFBSs of SP1 act as the crucial transcription factor that remains active and plays a significant role in essential biological mechanisms like cellular organization. The diversity and richness of these datasets provide a solid foundation for testing the effectiveness of our algorithm.

#### Dataset Pre-Processing

In processing the attC dataset, we focused on enhancing the dataset by removing irrelevant features, specifically those with lower variance. We identified and eliminated a total of 14 features that did not contribute meaningfully to our analysis, as low variance features are characterized by having over 80% of their values being the same. After this filtration, 278 features were left. After repeatedly testing different values for the recombination rate threshold, we ultimately determined that a threshold of 0.46 provides the best segmentation of our dataset, based on comparative experimental results. We then normalized these feature values linearly to a range between 0 and 1, which allowed us to introduce a new feature column that indicates whether a sample is negative or positive based on its recombination frequency: samples exceeding the threshold are classified as positive (marked as 1), while those below are classified as negative (marked as 0). This process resulted in an aggregate of 1,762 true instances and 11,117 false instances. To ensure a balanced dataset, we employed oversampling techniques, replicating the positive samples through replacement sampling to yield other samples, ultimately creating a balanced dataset of 22,234 samples.

**Fig. 3:**
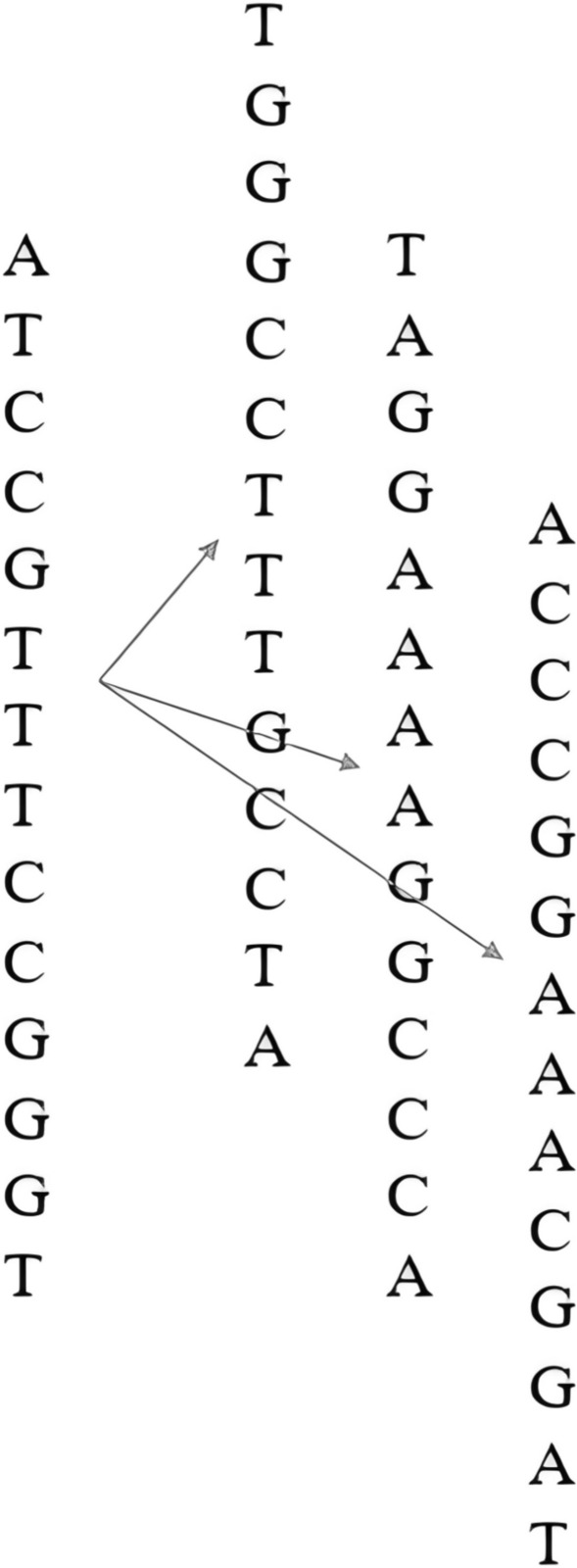
Examples of the data augmentation process

Regarding the TFBS dataset, we tackled the issue of limited DNA sequence samples by expanding the dataset through augmentation. As illustrated in Fig. 3, we generated additional sequences by calculating the reverse, complement, and reverse complement of each original DNA sequence. These newly created sequences were treated as additional data, enhancing our understanding of DNA–protein interactions. The labels for these augmented sequences remained consistent with those of the original samples. Consequently, the count of true instances increased from 1200 to 4800, while the negative samples also expanded accordingly.

**Fig. 4:**
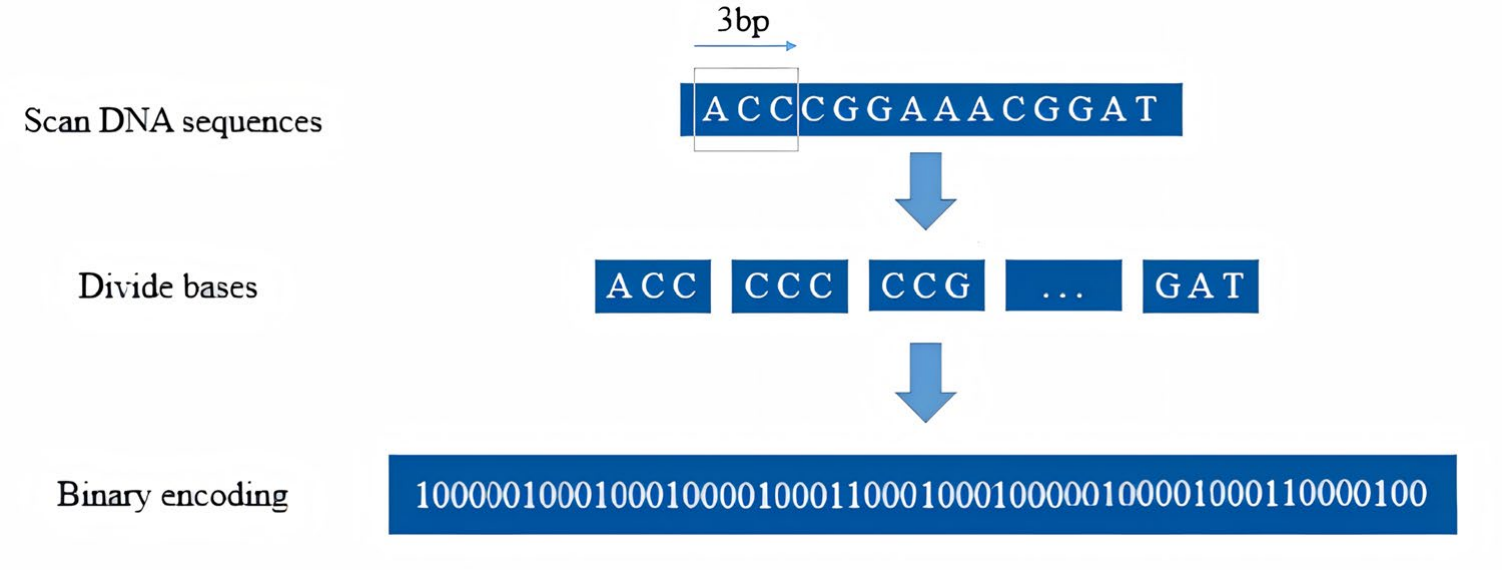
The details of multi-base feature encoding

**Fig. 5:**
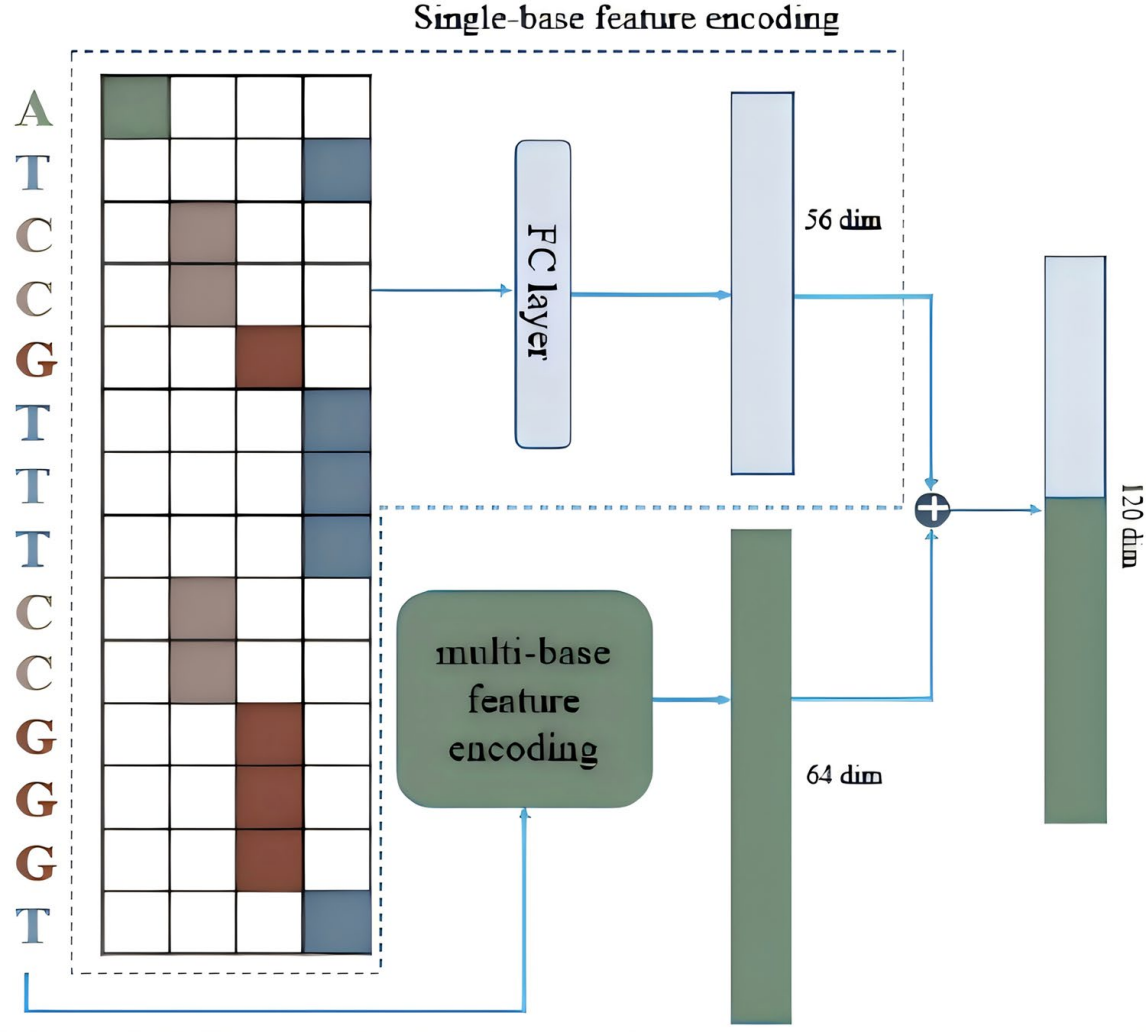
Workflow chart of multi-base feature encoding

For encoding the DNA sequences, we utilized a hybrid approach that combined one-hot encoding with multi-base encoding. In this method, the two binary encoded sequences were concatenated to form a final feature vector. The one-hot encoding process is detailed in Table 3, resulting in a length of 56 for each 14-base DNA sequence. For multi-base feature encoding, we associated each nucleotide with its surrounding context. On the other hand, we also need to address the issue of choosing the window size for encoding. The triplet corresponds to the encoding of an amino acid, which aligns with the basic units of the genetic code. Therefore, we set the sliding window size for encoding to 3, as it can more directly reflect biological significance and help capture information related to biological functions and structures. As depicted in Fig. 4, the approach constructs 56 trait attributes by grouping the DNA sequence into sets of three bases, applying a sliding window approach in increments of 1. Each trait attributes associated with a triplet within the window is marked with 1. Ultimately, the count of 16-base trait achieved 64. We combined the outputs from both encoding techniques to obtain 120 features in Fig. 5, integrating the results to enhance the dataset's comprehensiveness.

**Table 3:** One-hot encoding rules for nucleotides

Nucleotides	Binary Equivalent
A	1000
T	0100
C	0010
G	0001

Table 4 illustrates the quantity of dataset prior and following pre-process. The dataset acquisition and processing methods are explained in the preceding text. For example, the number of positive samples, 1762, is derived from the reorganization and division of the dataset. A stochastic approach was utilized to select the training and validation groups at a 4:1 proportion.

**Table 4:** The number of DNA structures and known TFBSs used in attCr0 and TFBS datasets

Dataset	Status	Positive sites	Negative sites
attC	Before preprocessing	1762	11,117
	After preprocessing	11,117	11,117
TFBS	Before preprocessing	1200	1200
	After preprocessing	4800	4800

### Description of the Algorithms

Algorithms in the field of machine learning have strong plasticity, so different algorithms can be integrated and optimized. Improved performance can be realized by the optimized algorithm through the use of each algorithm. Random Forest is a relatively classic classification algorithm, composed of many kinds of decision trees, and the outcome is the category in which decision trees account for the majority [30]. Through the optimization of Random Forest using weighted varied granularity scanning, the higher performance is achieved than Deep Forest. Deep Forest is an emerging deep learning method primarily applied for different tasks. Unlike traditional deep learning methods, Deep Forest combines the advantages of decision trees and ensemble learning. The XGBoost algorithm [31, 32] is an improvement to the GBDT algorithm [33]. The underlying principle of GBDT is ensemble learning. XGBoost also integrates many decision trees and makes up of numerous weak classifiers to establish a strong classifier [34].

In our approach, Deep Forest effectively integrates the varied granularity scanning approach. Initially, all features are treated equally during the varied granularity scanning stage. However, it is crucial to prioritize specific features that significantly impact the results to boost predictive accuracy. To address this, we incorporate a weighted varied granularity scanning method. By constructing decision trees, we assess the importance of each feature and pick the one that has the lowest Gini coefficient to serve as the criterion for making splits. Thus, calculating the Gini index for each node is essential during model construction, as detailed in Eq. (1).

1 $$G_\text{node} = 1 - \left( {\frac{{N_\text{positive} }}{N}} \right)^{2} - \left( {\frac{{N_\text{negative} }}{N}} \right)^{2}$$

In this methodology, *N*_positive_ and *N*_negative_ represent the counts of nodes for categories “1” and “0”, respectively, while *N* suggests the total sum of training instances. The score for each node is computed using Eq. (2), incorporating the Gini indices *G*_node,0_ and *G*_node,1_. Next, the significance value in the weight vector has been calculated according to Eq. (3), where *S*_node_*(t)* reflects the importance of a feature in the decision tree and *T* is the count of trees. At last, the weight is measured through the normalization of the trait scores as outlined in Eq. (4), resulting in a vector that effectively captures the significance of each feature in the predictive model. This process enhances the robustness of the algorithm by ensuring that features are evaluated based on their contributions to overall performance.

2 $$S_\text{node} = G_\text{node} - G_{\text{node},0} - G_{\text{node},1}$$

3 $$I_{i} = \sum\limits_{t = 1}^{T} {S_\text{node} (t)}$$

4 $$W_{i} = \frac{{I_{i} }}{{\sum\nolimits_{j=1}^{d} {I_{j} } }}$$

**Fig. 6:**
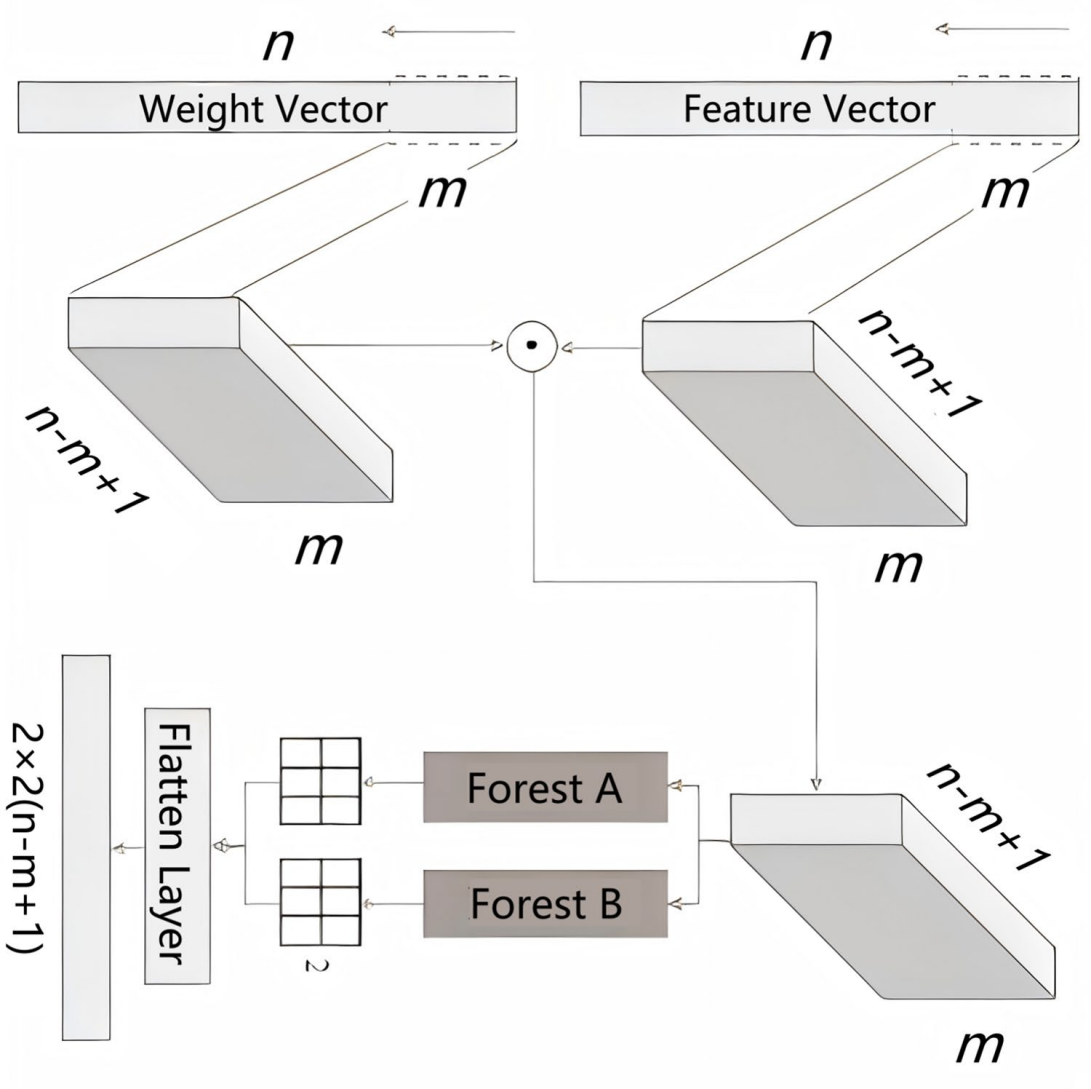
Workflow chart of the varied granularity scanning method

Using the varied granularity scanning approach, we are able to grasp the relationship among features and weights. The main computational process of this varied granularity scanning approach has been illustrated by Fig. 6. To begin, the moving frame of length *m* will be applied as it moves across both *F*(*n*), which represents the feature array. In this context, *n* denotes the total number of 0 or 1 represented by the base encodings. The window advances by a step size of 1, which allows for a thorough analysis of the distributions within the feature and weight vectors. Then, we obtain feature matrix $\varvec{f}^{(n-m+1,m)}$ and weight matrix $\varvec{w}^{(n-m+1,m)}$. Perform a Hadamard product of these two matrixs to obtain a pre-input value ***f***_pre_^(*n−m*+1, *m*)^, as shown in Eq. (5).

5 $$\varvec{f}_\text{pre}^{(n - m + 1,m)} = \varvec{f}^{(n - m + 1,m)} \odot \varvec{w}^{(n - m + 1,m)}$$

The pre-processed matrix ***f***_pre_^(*n−m*+1, *m*)^ is subsequently fed into both the complete deep forest and the standard deep forest model. Following this process, the models generate two distinct feature matrices, denoted as ***f***_*a*_^(*n−m*+1, 2)^ and ***f***_*b*_^(*n−m*+1, 2)^, respectively. Finally, these two feature matrices are combined by concatenating them into a unified one-dimensional feature vector ${{\varvec{F}}}^{(2\times 2\left(n-m+1\right))}$, which serves as the final representation for further processing or analysis.

**Fig. 7:**
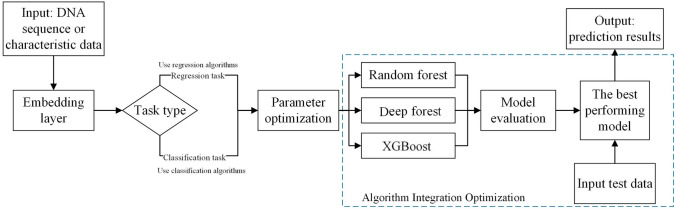
Workflow chart of OptimDase

Rooted in the algorithm integration, we use decision optimization to construct OptimDase. OptimDase combines and reconstructs the above algorithms to find the most capable algorithm for each dataset. Figure 7 displays the procedure of OptimDase.

OptimDase achieves site identification by learning DNA structural features. OptimDase requires three inputs: the preprocessed DNA binding site dataset *D'*, the test set *Z* which need to be predicted, and a flag (Flag) that indicates the task's sort. The prediction results for the test set constitute the output of OptimDase. OptimDase integrates and optimizes Random Forest, XGBoost, and Deep Forest algorithms. The description of OptimDase is as follows: first, it performs data pre-processing on the original dataset *D* as described in Sect. 4.1.2 to get data *D'* and determines a task's sort in line with a label flag. For example, classification algorithms are used in case that this task involves classification. Then, we chose the Optuna framework to optimize the hyperparameters of these three models. It performs a cyclic search within the ranges, where optimal hyperparameter values may exist for dataset *D'*, and updates the search intervals based on the results from each cycle. The dataset *D'* is split, with 80% allocated for training and 20% for validation, and submitted to the previously mentioned algorithm to train, respectively. During the training process, it is important to mention that we incorporated the weighted varied granularity scanning approach into Deep Forest. Next, the calculation is performed by the method for determining the weight vector. The weighted varied granularity scanning technique then receives these two vectors to extract these training features. Ultimately, the prediction algorithm is achieved through the process of instructing the model using a deep forest approach. In accordance with the assessment criteria, the finest model will be considered an ultimate predicting model *M*. At last, the test set *Z* is input *M* for prediction. The pseudo-code of OptimDase is shown in Algorithm 1.

**Figure:**
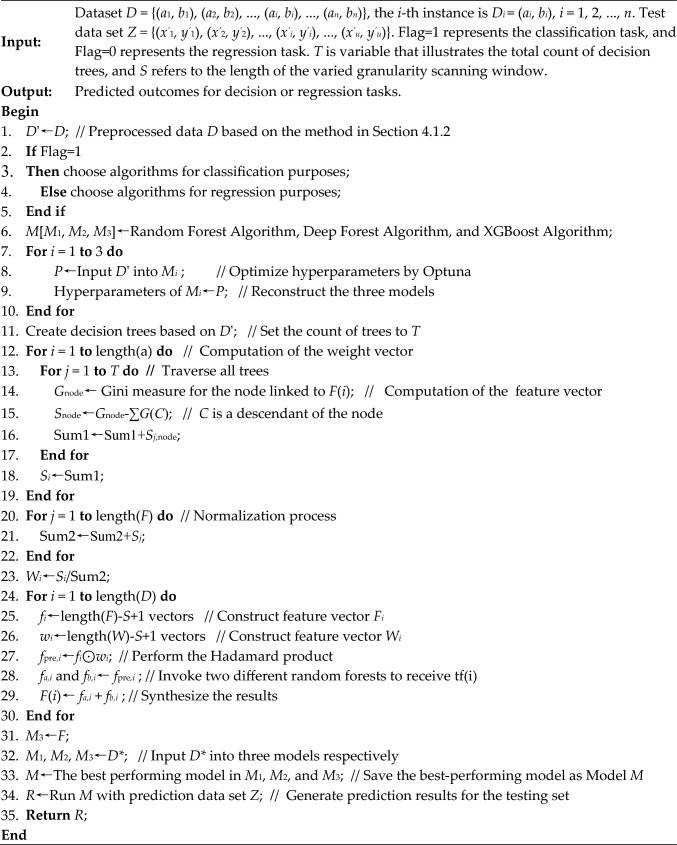
**Algorithm 1** OptimDase (*D*, Flag,* Z*)

### Training of the Algorithm

To ensure the scalability of the algorithms, they were developed and run on a Linux operating system. At the same time, most of the models used in this research were implemented using the Python Scikit-learn library, specifically version 3.6.19, which ensures compatibility with higher versions.

The hyperparameter tuning, which can greatly enhance model performance, is essential in machine learning. However, finding suitable hyperparameters can often be quite challenging. Manual selection tends to be time-consuming and labor-intensive, and the outcomes are usually suboptimal. Therefore, we opted for Optuna to adjust the hyperparameters to achieve optimal performance. Optuna is a user-friendly infrastructure for hyperparameter optimization. It conducts cyclic searches within the ranges where optimal hyperparameter values might exist, updating the search intervals based on the results from each cycle. We configured the number of searching loops to 800 and the optimized hyperparameters are presented in Table 5.

**Table 5:** Parameters used for other algorithms

Dataset	Algorithms	Parameters
attC	Random Forest	n_estimators = 695; max_depth = 18
	XGBoost	n_estimators = 1562; max_depth = 14; learning_rate = 0.028
	LightGBM	learning_rate = 0.2; max_depth = 1
	Deep Forest	default parameters
	OptimDase	Flag = 1
TFBS	AdaBoost	n_estimators = 50
	Random Forest	n_estimators = 462, max_depth = 13
	KNN	k = 3
	LightGBM	learning_rate = 0.0861, max_depth = 20
	Deep Forest	default parameters
	OptimDase	Flag = 0

OptimDase can be applied to general DNA binding site identification. At the same time, we exploited Optuna to explore different hyperparameters and tried to simplify the model design, which is convenient to contrast with the other models. Our model is mainly aimed at small datasets because many existing models are prone to overfitting when dealing with small datasets.

### Performance Evaluation

To assess the effectiveness of the algorithm in the classification process, we employed some evaluation metrics: accuracy, F1-score, and AUC. The metrics are derived from a confusion matrix comparing the actual values with the predicted values. Within the matrix of confusion, *n*_TP_, *n*_TN_, *n*_FP_, and *n*_FN_ denote the count of true positives, true negatives, false positives, and false negatives, respectively.

Equation (6) illustrates accuracy, which can be characterized as the relationship between the number of correct predictions and the total predictions made by the algorithm. This metric ranges from 0 to 1, indicating the frequency of correct classifications.

6 $$\text{Accuracy} = \frac{{n_\text{TP} + n_\text{TN} }}{{n_\text{TP} + n_\text{TN} + n_\text{FP} + n_\text{FN} }}$$

F1-score serves as an essential metric for evaluating the performance of the model. As outlined in Eq. (9), it is derived from both recall and precision. Recall pertains to our original dataset, reflecting the fraction of actual positive instances within the test data that have been accurately identified. On the other hand, precision assesses the reliability of our predictions, indicating the fraction of positive predictions that are true positives. Equations (7) and (8) define precision and recall, respectively.

7 $${\text{Recall}} = \frac{{n_\text{TP} }}{{n_\text{TP} + n_\text{FN} }}$$

8 $$\text{Precision} = \frac{{n_\text{TP} }}{{n_\text{TP} + n_\text{FP} }}$$

9 $$\text{F1-score} = \frac{{2 \times {\text{Recall}} \times \text{Precision}}}{{{\text{Recall}} + \text{Precision}}}$$

To create the ROC curve, the true positive rate is plotted on the vertical axis, while the false positive rate is plotted on the horizontal axis. The zone bounded by the axis lines beneath the ROC curve is the AUC value, which provides an objective measure of the algorithm's performance.

In the course of our regression analysis study, we utilized some evaluation metrics to judge the model: RMSE, PCC, and VarScore. Defined in Eq. (10), RMSE measures the discrepancy between the predicted values and the actual values, serving as a measure of their distance from one another. Of these, ${y}_{i}$ and ${y}^{\prime}_{i}$ denote the actual value and the prediction.

10 $$\text{RMSE} = \frac{1}{n}\sum\limits_{i = 1}^{n} {\sqrt {(y_{i} - y^{\prime}_{i} )^{2} } }$$

PCC illustrates the relationship among the predicted results and their actual counterparts. The method for calculating this metric is detailed in Eq. (11). In this eduation, ${\overline{y} }_{i}$ and $\overline{y }_{i}^{\prime}$ reflects the average result of the actual value and the prediction.

11 $$\text{PCC} = \frac{{\sum\nolimits_{i = 1}^{n} {(y_{i} - \overline{y}_{i} )(y^{\prime}_{i} - \overline{y}^{\prime}_{i} )} }}{{\sqrt {\left[ {\sum\nolimits_{i = 1}^{n} {(y_{i} - \overline{y}_{i} )^{2} } } \right]} \left[ {\sum\nolimits_{i = 1}^{n} {(y^{\prime}_{i} - \overline{y}^{\prime}_{i} )^{2} } } \right]}}$$

Defined in Eq. (12), VarScore evaluates how strongly dataset fluctuations affect the algorithm. The function *V*(·) represents the calculation of variance.

12 $$\text{VarScore} = \frac{1}{n}\left( {1 - \frac{{V(y_{i} - y^{\prime}_{i} )}}{{V(y_{i} )}}} \right)$$

## Conclusions

In computational biology, identifying DNA binding sites has always become a significant project. In the study, we introduce an algorithm called OptimDase, which utilizes combined feature encoding and optimum decision-making to predict DNA binding sites. Through fivefold cross-validation, OptimDase achieved accuracy, F1-score, and AUC of 0.8943, 0.8920, and 0.9219, for predicting DNA binding sites. Additionally, the algorithm demonstrated scores of 0.8721 for PCC, 0.0391 for MAE, 0.0054 for RMSE, and 0.7664 for VarScore in predicting the frequency of DNA recombination. During the research process, we built the model using the Python programming language on the Linux platform, ensuring that OptimDase has strong robustness and portability. The study indicates that the capability of our model in predicting DNA binding sites notably outperforms existing machine learning methods.

## Data Availability

We declare that the DNA binding sites data in the paper have been used with permission from Kaggle.com.
